# Temporal changes in occupational sitting time in the Danish workforce and associations with all-cause mortality: results from the Danish work environment cohort study

**DOI:** 10.1186/s12966-015-0233-1

**Published:** 2015-06-02

**Authors:** Hidde P. van der Ploeg, Simone Visbjerg Møller, Harald Hannerz, Allard J. van der Beek, Andreas Holtermann

**Affiliations:** Department of Public and Occupational Health, EMGO Institute for Health and Care Research, VU University Medical Center Amsterdam, van der Boechorststraat 7, NL-1081 BT Amsterdam, The Netherlands; National Research Centre for the Working Environment, Amsterdam, Denmark

**Keywords:** Sitting, Mortality, Workforce, Prospective cohort

## Abstract

**Background:**

Prolonged sitting has been negatively associated with a range of non-communicably diseases. However, the role of occupational sitting is less clear, and little is known on the changes of occupational sitting in a working population over time. The present study aimed to determine 1) temporal changes in occupational sitting time between 1990 and 2010 in the Danish workforce; 2) the association and possible dose-response relationship between occupational sitting time and all-cause mortality.

**Methods:**

This study analysed data from the Danish Work Environment Cohort Study (DWECS), which is a cohort study of the Danish working population conducted in five yearly intervals between 1990 and 2010. Occupational sitting time is self-reported in the DWECS. To determine the association with all-cause mortality, the DWECS was linked to the Danish Register of Causes of Death via the Central Person Register.

**Results:**

Between 1990 and 2010 the proportion of the Danish workforce who sat for at least three quarters of their work time gradually increased from 33.1 to 39.1 %. All-cause mortality analyses were performed with 149,773 person-years of observation and an average follow-up of 12.61 years, during which 533 deaths were registered. None of the presented analyses found a statistically significant association between occupational sitting time and all-cause mortality. The hazard ratio for all-cause mortality was 0.97 (95 % CI: 0.79; 1.18) when ≥24 hr/wk occupational sitting time was compared to <24 hr/wk for the 1990–2005 waves.

**Conclusions:**

Occupational sitting time increased by 18 % in the Danish workforce, which seemed to be limited to people with high socio-economic status. If this increase is accompanied by increases in total sitting time, this development has serious public health implications, given the detrimental associations between total sitting time and mortality. The current study was inconclusive on the specific role that occupational sitting might play in the increased all-cause mortality risk associated with the total volume of sitting.

## Introduction

The health risks of prolonged sitting time are becoming increasingly evident [1–4]. Large epidemiological studies have shown that total sitting time is associated with increased risk of all-cause, cardiovascular and possibly cancer mortality [5–7]. A recent meta-analysis suggests that the association between total sitting time and all-cause mortality is not linear and risks start to increase more steeply around 7 or 8 h of self-reported sitting time per day [1].

Less is still known about the different types and domains of sitting time and the association with disease and mortality risk. Television viewing has been studied most extensively and has shown the strongest associations for the risk of developing type 2 diabetes and cardiovascular disease as well as for mortality [8]. The association between health and other domains of sitting time, such as during work and transportation, are less well studied and often suffer from methodological limitations, most notably around measurement [9].

Occupational sitting time is often assessed through questioning participants about their predominant activity at work (sitting, standing, walking, heavy labour). The current study is one of few with a more continuous measure of occupational sitting time not entangled with occupational moderate to vigorous physical activity, which makes it possible to better study the independent effect of occupational sitting time.

Sitting behaviour is considered to have changed substantially over the last century due to the automation and computerization of many processes in daily life. However, few studies to date have reported on population changes in sitting time based on a representative sample. Time use data from the Netherlands suggests that the estimated proportion spent sitting remained relatively constant around 60 % of non-occupational time between 1975 and 2005 [10]. A similar trend was observed for leisure time, but the estimated proportion spent sitting was 85 % of leisure time in Dutch adults. Australian time use data estimated this was 90 % of leisure time in Australian adults [11]. Due to methodological limitations, time use data is less suitable for estimating the proportion spent sitting during occupational time. Crude estimates from the US suggest an increase in sedentary jobs from approximately 15 to 25 % of all jobs from 1960 to 2010 [12]. Australian National Health Survey data from 2007/2008 indicate that proportions of sedentary jobs might be higher, with 42 % of men and 47 % of women reporting they were mostly sitting during working hours, but no time trend data were available [13]. The current study has the ability to study temporal trends on occupational sitting time in a representative sample of the Danish workforce with five time points spread over two decades.

Hence, the current study aimed to determine: 1) temporal changes in occupational sitting time between 1990 and 2010 in the Danish workforce; and 2) the association and possible dose-response relationship between occupational sitting time and all-cause mortality.

## Methods

### Study population

This study analysed data from the Danish Work Environment Cohort Study (DWECS). DWECS is a cohort study of the Danish working population conducted in five yearly intervals between 1990 and 2010. The dynamic cohort has a split panel design consisting of a main panel randomly drawn from Danish residents aged 18–59 years in October 1990, and additional age and migration panels drawn at all other waves (1995–2010). The additional panels were included to adjust for migration and low response rates among young people in order to ensure representativeness of the Danish working population. In 2005 and 2010, the main cohort drawn in 1990 was supplemented with new participants randomly drawn from Danish residents. Once a subject was drawn for a certain panel and invited for the study he/she was re-invited at all following waves, irrespective of participation at previous waves. Participants enter the cohort when participating in the first interview.

Data was collected by telephone interviews in the 1990–2000 waves, by either postal or web-based questionnaires (90 %) or telephone interviews (10 %) in 2005, and by web-based questionnaires in 2010 [14]. Response rates varied from 90 % in 1990 to 53 % in 2010. For the temporal change in occupational sitting time, all five available waves of the DWECS (1990–2010) were used. For the analyses of the association between occupational sitting time and all-cause mortality risk, the 1990, 1995, 2000 and 2005 waves were used. The DWECS was approved by the Danish Data Protection Agency, journal numbers 2007-54-0059 and 2012-54-0042. More details on the DWECS can be found elsewhere [15].

### Study variables

Occupational sitting time was assessed in the DWECS using the question ‘Does your job involve sitting?’ with pre-set answer categories: ‘Almost all of the time; approximately ¾ of the time; approximately ½ of the time; approximately ¼ of the time; rarely; never’. This question is similar to a total sitting time question utilized successfully in earlier epidemiological studies [5]. The occupational sitting time question was combined with self-reported actual working hours in main and secondary jobs in order to estimate actual time spent sitting at work. To accomplish this, total actual weekly working hours were multiplied by the coefficients 0.875, 0.75, 0.5, 0.25, 0.125, and 0 for the ‘almost all of the time, ¾ of the time, ½ of the time, ¼ of the time, rarely, and never’ answering categories, respectively.

To determine the association with all-cause mortality, the DWECS was linked to the Danish Register of Causes of Death via the Central Person Register. The Central Person Register contains information on gender, addresses and dates of birth, death and migration for every person who is or has been an inhabitant of Denmark sometime since 1968 [16]. Adult DWECS participants (≥21 years) with a body mass index (BMI) between 18.5 and 50 who were categorised as employees during DWECS-interview in 1990 (October-November) entered our mortality follow-up on 1 January 1991 if they were still alive and living in Denmark on that date. Participants who entered the DWECS in 1995, 2000, or 2005 were followed for the all-cause mortality analyses from the first of January of the ensuing year. Included DWECS participants were followed until any of the following events occurred: the participant died, emigrated, or the analysis censor date was reached (31 December 2010).

Potential confounders of the association between occupational sitting time and all-cause mortality assessed in the DWECS were age, gender, socio-economic status, self-rated health, BMI, smoking, leisure time physical activity, and fruit and vegetable consumption. Socio-economic status was classified according to employment grade, job title and education into five social classes (I-V) [17]. Social class I: executive managers and/or having university a degree. Social class II: middle managers and/or 3–4 years of higher education. Social class III: Other white-collar workers. Social class IV: Skilled blue-collar workers. Social class V: Semi- or unskilled blue-collar workers. Socio-economic status was dichotomized into low (IV-V) and high (I-III) for stratification.

Self-rated health was assessed with a one-item questionnaire using five answering categories from the 36-Item Short Form Health Survey [18], which were dichotomized into poor (poor and very poor answering categories combined) and good health (fair, good and very good answering categories combined). Body height and weight were self-reported and used to calculate BMI, which was grouped into normal (18.5–24.9), overweight (25–29.9) and obese (≥30–50) following World Health Organization criteria [19]. Participants self-reported their smoking status as never, former or current smoker. Leisure time physical activity was assessed by questionnaire in 2000 and 2005 only and with slightly different questions in those two waves. For standardization purposes between the 2000 and 2005 waves, leisure time physical activity was dichotomized into those participants with less or those with more than 2 h of light intensity (>2 MET) physical activity per week. Fruit and vegetable consumption was self-reported in 2000 and 2005 only. This was dichotomized into those consuming fruit or vegetables at least once a day and those with less frequent consumption [20].

### Statistical analyses

The analysis plan was posted online prior to the analysis performed (http://figshare.com/articles/Study_protocol_The_associations_between_sitting_at_work_and_all_cause_mortality/980714). Respondents with missing data on occupational sitting or confounder variables at all waves were excluded from the analyses (*n* = 234). In case of a missing data point for an exposure or confounder variable at a certain wave, the most recently available information was carried forward.

Temporal changes in occupational sitting time in the Danish workforce between 1990 and 2010 were explored with descriptive statistics. Crude as well as age and gender standardised proportions were calculated, for which the 1990 DWECS wave was used as the reference. Stratified analyses by socio-economic status were carried out in order to determine if results were biased by the decline in response rate over the concurrent measurement waves.

Poisson proportional hazards regression was used to estimate hazard ratios (HR) and corresponding 95 % confidence intervals for the mortality risk in sedentary workers (occupational sitting time ≥24 hr/wk) compared to less sedentary workers (occupational sitting time <24 hr/wk). The primary analysis included all four waves (1990–2005) with occupational sitting time dichotomized at 24 hr/wk and adjusted for age, gender, socio-economic status and calendar year. A further similar analysis added adjustments for BMI, smoking, leisure time physical activity, and fruit and vegetable consumption, but this analysis only included the 2000 and 2005 waves due to the absence of leisure time physical activity and fruit and vegetable consumption assessments in the earlier waves. In order to determine possible reverse causality due to latent disease, three sensitivity analyses were performed that further adjusted for self-rated health; excluded participants with poor self-rated health at study entry; and excluded cases of death occurring within 3 years of cohort entry. Also, a sensitivity analysis was performed excluding participants under the age of 40, who are most likely presented with etiologies not likely to be associated with sitting time (such as head trauma). Since the used threshold of occupational sitting time was rather arbitrary (24 hr/wk), two further sensitivity analyses were performed by changing the threshold to 18 and 30 hr/wk, respectively.

In order to test a possible dose-response relationship between occupational sitting time and all-cause mortality, the same set of analyses was then repeated with occupational sitting time entered into the model as a continuous variable. Furthermore, a Χ^2^ goodness of fit test was conducted to determine a possible dose-response relationship. For the goodness of fit test, an ordinal scale was used for occupational sitting time (0; >0- < 10; 10- < 20; 20- < 30; 30- < 35; ≥35 hr/wk). All analyses were performed in SAS version 9.3. The statistical significance threshold was set at p < 0.05.

## Results

Table 1 presents the characteristics of the DWECS participants for all five waves between 1990 and 2010. The study sample has an equal gender balance and reflects well known societal trends with regard to the ageing of the workforce, the increasing prevalence of overweight and obesity, and the decrease in smoking prevalence.

**Table 1:** Characteristics of participants in the Danish Work Environment Cohort Study 1990-2010

	1990		1995		2000		2005		2010	
	n	%	n	%	n	%	n	%	n	%
Total	5608	100	5238	100	5926	100	8769	100	10624	100
Age (years)										
21–40	3078	54.9	2726	52.0	2826	47.7	3401	38.8	3410	32.1
40–49	1570	28.0	1469	28.0	1567	26.4	2595	29.6	3323	31.1
50–59	960	17.1	922	17.6	1274	21.5	2453	28.0	3242	30.5
60–69	0	0	121	2.3	259	4,4	306	3.5	649	6.1
≥ 70	0	0	0	0	0	0	14	0.2	0	0
Gender (women)	2708	48.4	2485	47.4	2804	47.3	4462	50.9	5571	52.4
Socio-economic status^a^										
social class I (highest)	715	12.7	711	13.6	898	15.2	1537	17.5		
social class II	1045	18.6	800	15.3	1250	21.1	2262	25.8		
social class III	1997	35.6	1912	36.5	1762	29.7	2004	22.9		
social class IV	555	9.9	596	11.4	705	11.9	1315	15.0		
social class V (lowest)	1140	20.3	1041	19.9	1056	17.8	1279	14.6		
missings	156	2.8	178	3.4	155	4.3	372	4.3		
Self-rated health										
fair, good, or very good	5528	98.6	5157	98.5	5815	98.1	8538	97.4	9963	93.8
very poor or poor	79	1.4	77	1.5	101	1.7	180	2.1	322	3.0
missings	1	0.0	4	0.1	10	0.2	51	0.6	339	3.2
BMI (kg/m^2^)										
normal (18.5 - < 25)	4006	71.4	3474	66.3	3520	59.4	4806	54.8	5365	50.5
overweight (25- <30)	1317	23.5	1437	27.4	1888	31.9	2909	33.2	3640	34.3
obese (30+)	260	4.6	290	5.5	457	7.7	901	10.3	1339	12.6
missings	25	0.4	37	0.7	61	1.0	1.7	1.7	280	2.6
Smoking										
never	1954	34.8	1961	37.4	2372	40.0	3807	43.4	5007	47.1
former	1006	17.9	1078	20.6	1355	22.9	2390	27.3	3069	28.9
current	2648	47.2	2198	42.0	2190	37.0	2515	28.7	2332	22.4
missings	0	0	1	0.0	9	0.2	57	0.7	216	2.0
Leisure time physical activity (<2 h/wk of light intensity)^a^										
No					5015	84.6	7262	82.8		
Yes					895	15.1	1427	16.3		
missings					16	0.3	80	0.9		
At least daily fruit or vegetable consumption										
Yes					4326	73.0	6721	76.6	7930	74.6
No					1588	26.5	2019	23.0	2213	20.8
missings					12	0,2	29	0,3	481	4.5

Variables without a ‘missings’ row had no missing values

^a^In 2010, the variable was assessed quite differently and hence excluded from the table

### Temporal changes in occupation sitting time

Table 2 shows the changes in occupational sitting time in the Danish workforce over the five waves between 1990 and 2010. The proportion of the Danish workforce who sat for at least three quarters of their work time gradually increased from 33.1 % in 1990 to 39.1 % in 2010. The stratified analysis revealed an increase in occupational sitting time for people with high but not low socio-economic status.

**Table 2:** Changes in occupational sitting time in the Danish workforce between 1990–2010

Year	1990	1995	2000	2005	2010
Total population					
Population	5933	5554	6102	9037	10986
Percentage sitting ≥ 3/4 of work time	33.1	32.4	34.1	36.9	40.1
Percentage sitting ≥ 3/4 of work time, age & gender standardized (95 % CI)	33.1 (32.0; 34.3)	32.3 (31.1; 33.6)	33.5 (32.3; 34.7)	35.3 (34.3; 36.4)	39.1 (38.1; 40.2)
Low socio-economic status					
Population	2068	2003	2065	2927	NA
Percentage sitting ≥ 3/4 of work time	17,0 %	16,7 %	16,8 %	17,0 %	NA
Percentage sitting ≥ 3/4 of work time, age & gender standardized (95 % CI)	17,0 % (15.5; 18.7)	16,7 % (15.1; 18.4)	16,7 % (15.1; 18.4)	16,6 % (15.2; 18.2)	NA
High socio-economic status					
Population	3814	3461	3940	5878	NA
Percentage sitting ≥ 3/4 of work time	41,9 %	41,8 %	43,2 %	46,8 %	NA
Percentage sitting ≥ 3/4 of work time, age & gender standardized (95 % CI)	41,9 % (40.4; 43.5)	41,9 % (40.3; 43.6)	43,3 % (41.7; 45.0)	46,4 % (45.0; 47.8)	NA

*NA* not available for 2010 as socio-economic status was assessed differently compared to the previous measurement waves

### Occupational sitting time & all-cause mortality

The analyses that utilized the four waves from 1990 to 2005 included 149,773 person-years of observation (mean [SD] follow-up time, 12.61 [6.76] years) during which 533 deaths were registered. The analyses of 2000 & 2005 waves only included 73,297.67 person-years of observation (mean [SD] follow-up time, 7.47 [2.56] years) during which 172 deaths were registered. The results from the Poisson proportional hazards regression analyses are presented in Tables 3 and 4, where occupational sitting time was entered as dichotomized and continuous variables, respectively. Both tables also present the results from the analyses from the 1990–2005 waves and those from the 2000 & 2005 waves that included more adjustments. None of the presented analyses found a statistically significant association between occupational sitting time and all-cause mortality. The HR was 0.97 (95 % CI: 0.79; 1.18) when ≥24 hr/wk occupational sitting time was compared to <24 hr/wk for the 1990–2005 waves, while the HR was 1.25 (95 % CI: 0.90; 1.74) for the 2000 & 2005 waves that included more adjustments. Per 10 hr/wk increase in occupational sitting time the HRs were 0.97 (95 % CI: 0.91; 1.05) and 1.09 (95 % CI: 0.97; 1.23), respectively.

**Table 3:** Poisson proportional hazard ratios for all-cause mortality and occupational sitting time (≥24 hr/wk)

	All-cause mortality			
	Follow-up 1991–2010^a^		Follow-up 2001–2010^b^	
	HR	95 % CI	HR	95 % CI
Occupational sitting time				
<24 hr/wk	1.00	Reference	1.00	Reference
≥24 hr/wk)	0.97	0.79; 1.18	1.25	0.90; 1.74
Age (years)				
21–40	1.00	Reference	1.00	Reference
40–49	3.74	2.31; 6.06	4.36	2.00; 9.51
50–59	9.98	6.38; 15.61	9.07	4.32; 19.07
60–69	23.75	15.22; 37.07	21.45	10.22; 45.02
≥70	52.01	32.14; 84.16	58.79	23.80; 145.24
Gender				
women	1.00	Reference	1.00	Reference
Men	1.62	1,35; 1.94	1.43	1.02; 20.00
Socio-economic status				
social class I (highest)	1.00	Reference	1.00	Reference
social class II	1.14	0.81; 1.60	1.36	0.76; 2.44
social class III	1.33	0.98; 1.80	1.61	0.95; 2.74
social class IV	1.23	0.85; 1.78	1.25	0.65; 2.38
social class V (lowest)	1.86	1.36; 2.53	1.94	1.11; 3.38
BMI (kg/m^2^)				
normal (<25)			1.00	Reference
overweight (25- <30)			0.83	0.59; 1.16
obese (30+)			0.86	0.52; 1.41
Smoking				
never			1.00	Reference
former			1.34	0.87; 2.06
current			2.01	1.36; 2.97
Leisure time physical activity (<2 h/wk of light intensity)				
No			1.00	Reference
Yes			1.38	0.95; 1.99
At least daily fruit or vegetable consumption				
No			1.00	Reference
Yes			0.61	0.44; 0.85

^a^Adjusted for age, gender, socio-economic status and calendar year

^b^Adjusted for age, gender, socio-economic status, BMI, smoking, leisure time physical activity and fruit and vegetable consumption

**Table 4:** Poisson proportional hazard ratios for all-cause mortality and occupational sitting time (as continuous variable)

	All-cause mortality			
	Follow-up 1991–2010^a^		Follow-up 2001–2010^b^	
	HR	95 % CI	HR	95 % CI
Occupational sitting time (per 10 hr/wk increase)	0.97	0.91; 1.05	1.09	0.97; 1.23
Age (years)				
21–40	1.00	Reference	1.00	Reference
40–49	3.74	2.31; 6.06	4.36	2.00; 9.49
50–59	9.98	6.38; 15.63	9.08	4.32; 19.09
60–69	23.69	15.18; 36.98	21.56	10.27; 45.35
≥ 70	51.67	31.92; 83.63	59.56	24.08; 147.30
Gender				
women	1.00	Reference	1.00	Reference
Men	1.63	1.35; 1.95	1.41	1,00; 1.98
Socio-economic status				
social class I (highest)	1.00	Reference	1.00	Reference
social class II	1.13	0.80; 1.58	1.37	0.76; 2.46
social class III	1.31	0.96; 1.78	1.64	0.96; 2.80
social class IV	1.19	0.81; 1.74	1.30	0.68; 2.52
social class V (lowest)	1.81	1.31; 2.49	2.02	1.15; 3.55
BMI (kg/m^2^)				
normal (<25)			1.00	Reference
overweight (25- <30)			0.83	0.59; 1.16
obese (30+)			0.86	0.52; 1.41
Smoking				
never			1.00	Reference
former			1.34	0.87; 2.05
current			2.00	1.36; 2.96
Leisure time physical activity (<2 h/wk of light intensity)				
No			1.00	Reference
Yes			1.37	0.95; 1.99
At least daily fruit or vegetable consumption				
No			1.00	Reference
Yes			0.61	0.44; 0.85

^a^Adjusted for age, gender, socio-economic status and calendar year

^b^Adjusted for age, gender, socio-economic status, BMI, smoking, leisure time physical activity and fruit and vegetable consumption

All sensitivity analyses and the goodness of fit test showed similar results, and found no significant association between occupational sitting time and all-cause mortality (data not shown). Since it could be argued that overcorrection might occur due to employment grade and job title possibly being strongly correlated with occupational sitting time, an additional, post-hoc sensitivity analysis adjusted for education rather than the socio-economic status variable (i.e. constructed from education combined with employment grade and job title) was performed. This post-hoc sensitivity analysis also showed similar results for ≥24 hr/wk of occupational sitting time (HR = 0.93, 95 % CI = 0.77-1.13).

## Discussion

The results of this study suggest that occupational sitting time gradually increased between 1990 and 2010 in the Danish workforce, but only in people with high socio-economic status. This is in line with a study that showed sedentary jobs have become more prevalent in the USA since the 1960 [12]. This increase in occupational sitting time has important implications for public health as occupational sitting time is a major contributor to total sitting time, which is associated to a range of non-communicable diseases.

The study did not show a statistically significant association between occupational sitting time and all-cause mortality. However, the analyses with the 2000 & 2005 waves, which made further adjustments for BMI, smoking, leisure time physical activity, and fruit and vegetable consumption, revealed some hints of a possible adverse association between occupational sitting time and all-cause mortality. It should be noted that power analyses as described in the a-priori online published analysis plan showed the study sample was just large enough to study all-cause mortality in the full sample. Hence, there was a risk that the analysis of the 2000 & 2005 waves would not be sufficiently powered to statistically reveal clinically relevant findings. Given the limitations in statistical power and possible residual confounding, as discussed in more detail in the limitations section below, the results of our analyses of the association between occupational sitting time and all-cause mortality are inconclusive.

A systematic review on the health risks of occupational sitting time, identified six prospective studies investigating the association between occupational sitting time and all-cause mortality [9]. It was reported that four of the six prospective studies found that occupational sitting was associated with an increased mortality risk, one study found no association, and one study found that sitting was associated with a decreased mortality risk [9]. However, it should be noted that the focus of these prospective studies was mostly on moderate to vigorous intensity physical activity during work, which was compared to sitting at work. The correlation between moderate to vigorous intensity physical activity and sitting is poor, which illustrates they are distinctly different behaviours. Hence, it is important to disentangle moderate to vigorous intensity physical activity and sitting time in epidemiological analyses. A more recent study that analysed five Health Survey for England and two Scottish Health Survey cohorts revealed that women with a standing/walking occupation had lower all-cause mortality risk (HR 0.68, 95 % CI 0.52–0.89) than women with a sitting occupation, but there was no association in men [21]. However, in this study the measure of occupational sitting time also focussed on the predominant activity (sitting, standing, walking), which made it difficult to disentangle the influence of moderate to vigorous intensity physical activity and sitting time.

Based on the literature to date, methodological limitations mainly regarding the assessment of occupational and other domain specific sitting time make it difficult to draw conclusions on the possible association between occupational sitting time and all-cause mortality. However, the association between total sitting time and all-cause mortality seems well established [1]. Hence, for adults for whom a large proportion of their sitting time comes during working hours, it seems that solutions around reducing occupational sitting time such as sit-stand or active workstations are a good way to contribute to the reduction of total sitting time [22]. However, there might be a difference in the health risks of sitting time in different domains, i.e. maybe passively watching television poses higher health risk than actively working behind a desk. This is partly supported by some data suggesting that the mortality risks of watching television are higher than those of total sitting time [6]. Determining the risks of occupational sitting time currently poses a complicated puzzle that is hampered by methodological limitations, but possibly also by the entanglement with socio-economic status. People with higher socio-economic status often have white collar professions that are more likely to involve sitting at work. Hence, higher socio-economic status is often associated with higher levels of occupational sitting, but also with other healthier lifestyles (such as less smoking, healthier diet, and more moderate to vigorous physical activity), which could offset some of the possible detrimental effects of occupational sitting. In other words, if other lifestyle risk factors cluster in people who sit less during work time, this could potentially obscure the effects of occupational sitting time if analyses are not adequately corrected for those other lifestyle risk factors.

### Strengths & limitations

The main strength in this study is the use of a sample that is representative for the Danish labor force over two decades. The five data points allow for studying temporal trends as well as subsequently following up participants for all-cause mortality. Furthermore, information on occupational sitting time and confounders were measured repeatedly during the follow-up period, reducing misclassifications related to changes in exposure status. The assessment of occupational sitting as a ordinal variable enabled the examination of a possible dose-response association and enabled the disentanglement of occupational sitting and moderate to vigorous physical activity, which has been a limitation in previous studies. A limitation of the present study is the relatively low statistical power preventing stratified analyses, for example by age or gender. As already discussed, low statistical power was also a limitation for the analyses that only included the 2000 & 2005 waves. The results for the 2000 & 2005 waves were far from statistical significance, nevertheless the reported HR point estimates (1,25 and 1.09 for dichotomized and ordinally scaled occupational sitting time, respectively) would have been clinically relevant had they been statistically significant. Another limitation is the risk for residual confounding. Most notably the lack of a measure of total sitting time in the DWECS might have confounded our findings. It is possible that the contribution of participant’s occupational sitting time to total sitting time varied greatly, i.e. a person with a high occupational sitting time might be less likely to sit much outside work than a person with low occupational sitting time. Hence, it seems important that future analyses correct for total sitting time in order to determine the role that occupational sitting time plays in the health risks of sitting. Future studies should aim to include objective assessments of total sitting time and total physical activity. In order to obtain domain specific information, an objective measure of sitting time could be supplemented with a diary of working hours allowing to also determine occupational sitting time [23], or supplemented with a brief domain-specific sitting questionnaire such as the Workforce Sitting Questionnaire, which estimates leisure, transport, occupational and total sitting time [24].

Furthermore, the measures of self-reported physical activity (leisure time only with a focus on light intensity) and diet (fruit and vegetable intake only) might be other sources of misclassification and unsufficient correction for confounding. The inclusion of both physical activity and diet measures were strengths of the study, but their absence in the 1990 and 1995 waves led to the earlier discussed issues around statistical power in some of the analyses.

Finally, the response rate declined over the concurrent measurement waves, and it is possible that selection bias might have influenced the temporal trends we observed in occupational sitting time. Nevertheless, the temporal trends were age and gender standardized. Results were not standardized for socio-economic status, as actual population changes in socio-economic status are likely to have occurred and would be highly correlated with changes in occupational sitting time. In order to determine if the reported increase in occupational sitting time was due to selection bias for socio-economic status induced by the decline in response rate over the measurement waves, we stratified the temporal trends in occupational sitting time by socio-economic status. This showed the temporal increase in occupational sitting time was only present in people of high socio-economic status. However, data for 2010 were missing in this stratified analysis, due to the different assessment of socio-economic status.

## Conclusion

Occupational sitting time increased by 18 % in the Danish workforce, which seemed to be limited to people with high socio-economic status. If this increase is accompanied by increases in total sitting time, this development has serious public health implications, given the known detrimental associations between total sitting time and disease specific and all-cause mortality. However, due to methodological limitations, the current study was inconclusive on the specific role that occupational sitting might play in the all-cause mortality risks of too much sitting. Future prospective cohort studies should aim to include objective as well as domain-specific measures of sitting time in order to unravel the complex associations between the different domains of sitting time and morbidity and mortality.
